# Latent traits of impulsivity and compulsivity: toward dimensional psychiatry

**DOI:** 10.1017/S0033291717002185

**Published:** 2017-08-14

**Authors:** S. R. Chamberlain, J. Stochl, S. A. Redden, J. E. Grant

**Affiliations:** 1 Department of Psychiatry, University of Cambridge, Cambridge, UK; 2 Cambridge and Peterborough NHS Foundation Trust, Cambridge, UK; 3 Department of Kinanthropology, Charles University in Prague, Prague, Czech Republic; 4 Department of Psychiatry & Behavioral Neuroscience, University of Chicago, Chicago, USA

**Keywords:** Addiction, cognition, compulsive, habit, impulsive

## Abstract

**Background:**

The concepts of impulsivity and compulsivity are commonly used in psychiatry. Little is known about whether different manifest measures of impulsivity and compulsivity (behavior, personality, and cognition) map onto underlying latent traits; and if so, their inter-relationship.

**Methods:**

A total of 576 adults were recruited using media advertisements. Psychopathological, personality, and cognitive measures of impulsivity and compulsivity were completed. Confirmatory factor analysis was used to identify the optimal model.

**Results:**

The data were best explained by a two-factor model, corresponding to latent traits of impulsivity and compulsivity, respectively, which were positively correlated with each other. This model was statistically superior to the alternative models of their being one underlying factor (‘disinhibition’) or two anticorrelated factors. Higher scores on the impulsive and compulsive latent factors were each significantly associated with worse quality of life (both *p* < 0.0001).

**Conclusions:**

This study supports the existence of latent functionally impairing dimensional forms of impulsivity and compulsivity, which are positively correlated. Future work should examine the neurobiological and neurochemical underpinnings of these latent traits; and explore whether they can be used as candidate treatment targets. The findings have implications for diagnostic classification systems, suggesting that combining categorical and dimensional approaches may be valuable and clinically relevant.

## Introduction

There is a growing realization that the careful elucidation and measurement of intermediate biological characteristics is central to refining and improving existing psychiatric classification and treatment. As highlighted in the NIMH Research Domain Criteria (RDoC) strategic plan, it is necessary to identify intermediate markers, cutting across related psychiatric disorders, and continuous with relevant markers of variability in the general population (Cuthbert & Insel, 2013). The concepts of impulsivity and compulsivity are highly relevant clinically, but also represent fruitful heuristics in the search for intermediate markers of psychiatric disease. Impulsivity refers to behaviors or actions that are inappropriate, premature, unduly thought out, risky, and that lead to untoward outcomes (Evenden, 1999). Compulsivity refers to a tendency toward repetitive, habitual actions, repeated despite adverse consequences (Robbins *et al.* 2012). It has been suggested that impulsivity and compulsivity might constitute opposite ends of a spectrum (Stein *et al.* 1993). However, impulsive and compulsive symptoms can co-occur within the same individual, hence the suggestion that they may be driven by common neurobiological processes (such as lack of top–down executive control, or ‘disinhibition’) (Chamberlain *et al.* 2005). Impulsivity–compulsivity constitutes one of several candidate dimensional models in psychiatry. Other key examples include internalizing (depression, generalized anxiety) *v.* externalizing (e.g. substance use, antisocial personality) symptoms (Khan *et al.* 2005); and depression *v.* mania (the ‘mood spectrum’) (McElroy *et al.* 1996). These different frames of references can be viewed as being partly related.

At the level of symptoms, impulsive behaviors are explicitly listed in the diagnostic criteria for attention-deficit hyperactivity disorder (ADHD), and several other conditions formally listed as ‘Impulse Control Disorders’ in the Diagnostic and Statistical Manual Version 5 (DSM-5) (American Psychiatric Association, 2013). Symptoms reaching formal diagnostic criteria for ADHD are evident in up to 7% of children and 2.5% of adults (Simon *et al.* 2009). Compulsivity is well represented in obsessive–compulsive disorder (OCD; intrusive thoughts and/or repetitive rituals) and in obsessive–compulsive personality disorder (OCPD; e.g. rigid, perfectionistic approach to life with reluctance to delegate) (American Psychiatric Association, 2013). Formal OCD affects 1–3% of the population, and OCPD up to 8% (Grant *et al.* 2014; Diedrich & Voderholzer, 2015). Obsessive–compulsive traits exist in milder forms and can be quantified using questionnaires designed for this purpose (Sanavio, 1988). Compulsivity is also a core behavioral feature in gambling disorder or substance use disorders – conditions now listed together in the DSM-5 category of ‘Substance Related and Addictive Disorders’. These disorders are also common (prevalence of 8.5% for alcohol use disorder and up to 3.1% for gambling disorder) (Grant *et al.* 2004*a*; Ferguson *et al.* 2011).

Impulsivity and compulsivity can be conceptualized not only in terms of overt psychopathology, such as described above, but also in terms of personality- and laboratory-based neurocognitive measures. There is an extensive literature on the development and validation of questionnaire-based measures of personality related to impulsivity (e.g. the Barratt Impulsiveness Questionnaire), and compulsivity (e.g. the Padua Obsessive–Compulsive Inventory) (Barratt, 1965; Sanavio, 1988). These measures are well suited for use at the level of the general population, but are also sensitive to more extreme levels of impulsivity and compulsivity as manifested in ADHD and OCD, respectively (Malloy-Diniz *et al.* 2007; van den Heuvel *et al.* 2008). From a cognitive perspective, facets of impulsivity can be captured using tests of premature motor response (stop-signal tasks), and gambling paradigms examining tendency for making risky decisions (Grant & Chamberlain, 2014). Similarly, aspects of compulsivity can be fractionated using tests of flexible responding, especially set-shifting (Robbins *et al.* 2012), and measures of rigidity on other tests. Inroads have been made in eliciting the neural and neurochemical substrates underlying normal performance on these tasks across species (Robbins, 2005; Dalley *et al.* 2011; Bari & Robbins, 2013; Fineberg *et al.* 2014). Dysfunction of these fronto-striatal systems is central to neurobiological models and treatment of impulsive and compulsive conditions (Denys *et al.* 2004; Biederman & Faraone, 2005; Del Campo *et al.* 2011; Seixas *et al.* 2012).

The concepts of impulsivity and compulsivity have contributed to the latest nosological classification systems in psychiatry, and are critical concepts when considering treatments for mental disorders. Yet, it is not yet known whether latent phenotypes of impulsivity and compulsivity exist; if so, whether they are related; and if so, how. Therefore, we quantified whether impulsive and compulsive data were optimally explained by there being one underlying latent factor, two underlying unrelated latent factors, or two underlying related latent factors. It was hypothesized that impulsive and compulsive variables would load onto two largely separable, but positively correlated latent traits.

## Materials and methods

### Participants

Participants, aged 18–29 years, were recruited using media advertisements in two US cities. Adverts asked subjects to participate in a research study exploring impulsive/compulsive behaviors. Subjects were excluded if they were unable to give informed consent, were unable understand/undertake the study procedures, or were seeking treatment for any mental disorders. All study procedures were carried out in accordance with the Declaration of Helsinki. The Institutional Review Boards of the Universities of Minnesota and Chicago approved the study and the consent statement. Participants were compensated with a $50 gift card for a local department store for taking part.

### Clinical assessments

Assessments were conducted in a quiet testing room with a trained rater, and included objective clinical interview, completion of questionnaires, and neuropsychological assessment using a touch-screen computer. An overview of the measures is provided in Table 1 below. We focused on measures of impulsivity and compulsivity, categorized as such *a priori* on the basis of current psychiatric models and nosology.

**Table 1.** Overview of outcome measures collected as part of the study

Measurement category	Instrument used	Description	References
Demographic			
Age	–	–	–
Gender	–	–	–
Education	–	–	–
Quality of life	Quality of Life Inventory (QOLI)	Quality of life *t*-score, based on responses to 32 items, encompassing well-being and life satisfaction	Frisch *et al.* (1992)
Impulsivity			
Psychopathology			
Attention-deficit hyperactivity disorder (ADHD) symptoms	World ADHD Rating Scale (ASRS v1.1), Part A	Gives total score, which indicates risk of underlying ADHD diagnosis	Kessler *et al.* (2005)
Suicidality	Mini International Neuropsychiatric Inventory (MINI)	Generates total score related to history of self-endangerment, suicidal thoughts, and suicide attempt(s). Score held to reflect current suicide risk	Sheehan *et al.* (1998)
Personality			
Antisocial personality disorder	MINI	Binary measure as to whether criteria met for antisocial personality disorder, based on structured clinical interview	Sheehan *et al.* (1998)
Motor, non-planning, and attentional impulsivity	Barratt Impulsiveness Questionnaire (BIS-11)	Yields three total sub-scores based on previous factor analysis	Barratt (1965), Patton *et al.* (1995)
Impulsiveness, venturesomeness, and extraversion	Eysenck Personality Inventory	Yields three total sub-scores based on previous factor analysis	Eysenck & Eysenck (1978), Eysenck & Eysenck (1977)
Cognition			
Response inhibition	CANTAB Stop-Signal Task (SST)	Estimates time taken for individual's brain to suppress a pre-potent response (stop-signal reaction time)	Aron *et al.* (2007), Logan *et al.* (1984)
Quality of decision-making	CANTAB Cambridge Gamble Task (CGT)	Measures tendency of individual to make impulsive decisions contrary to logic	Rogers *et al.* (1999)
Compulsivity			
Psychopathology			
Gambling disorder	Minnesota Impulse Disorders Inventory (MIDI) module	Binary presence/absence of gambling disorder, based on structured clinical interview	Grant *et al.* (2004*b*)
Gambling frequency	Structured interview	Person asked how many times they gamble in a typical week	–
Gambling severity	Pathological-Gambling Yale-Brown Obsessive-Compulsive Scale (PG-YBOCS)	Total score, based on thoughts and behaviors related to problem/pathological gambling	Pallanti *et al.* (2005)
Alcohol use disorder (AUD)	MINI	Binary measure as to presence or absence of AUD (dependence/abuse), based on structured clinical interview	Sheehan *et al.* (1998)
Alcohol frequency	Structured interview	Person asked how many times they consume one or more alcoholic beverages per week	–
Substance use disorder (SUD)	MINI	Binary measure as to presence or absence of SUD (dependence/abuse, any substance besides alcohol), based on structured clinical interview	Sheehan *et al.* (1998)
Marihuana consumption	In-person interview	Person asked how many times they consume cannabis per week	
Nicotine consumption	In-person interview	Person asked how much they smoke; responses converted to ‘packs per day’ equivalent	
Problematic use of the Internet	Young's Diagnostic Questionnaire	Gives total score as to how many criteria met for problematic Internet use (max 8)	Young (2009)
Eating disorder	MINI	Binary as to presence/absence of any eating disorder, based on structured clinical interview	Sheehan *et al.* (1998)
Personality			
Obsessive–compulsive (OC) symptoms	Padua Obsessive–Compulsive Inventory Revised	Thirty-nine-item questionnaire, which yields five scores for different OC factors	Burns *et al.* (1996), Sanavio, (1988)
Obsessive–compulsive personality disorder score	DSM-IV symptom tick-list	Total score as to how many of eight symptom criteria met	American Psychiatric Association (2000)
Cognition			
Extra-dimensional set-shifting	CANTAB Intra-Dimensional/Extra-Dimensional Set-Shift Task (IED)	Errors made during the crucial extra-dimensional shift stage of the test	Pantelis *et al.* (1999)
Reversal learning	CANTAB IED	Total number of errors made for all reversal learning stages of the test	Pantelis *et al.* (1999)
Risk adjustment	CANTAB CGT	Measures the extent to which participants modulate their behavior depending on risk level (flexible decision-making)	Rogers *et al.* (1999)

Psychopathological measures of impulsivity comprised ADHD symptom scores, occurrence of antisocial personality disorder (ASPD), and suicidal tendencies. Impulsive symptoms are listed explicitly in the diagnostic criteria for ADHD and ASPD, whereas many studies have reported strong – even heritable – associations between impulsivity and suicide-related behaviors (Chistiakov *et al.* 2012). For personality-related measures of impulsivity, we included the Barratt Impulsivity and Eysenck personality questionnaires, which are widely used and accepted for such purposes (Gomez & Corr, 2014; Stanford *et al.* 2016). For cognitive measures of impulsivity, we focused on the inhibition of pre-potent motor responses on the Stop-Signal Test (SST), and the tendency to make irrational decisions to the detriment of longer term performance on the Cambridge Gamble Test (CGT). Decisional and motor impulsivity are widely recognized as distinct cognitive manifestations of impulsivity (Dalley *et al.* 2011; MacKillop *et al.* 2016). See online Supplementary file for more detailed description of the cognitive tasks.

Compulsive measures of psychopathology included those reflecting gambling, substance use, compulsive use of the Internet, and eating disorders. Compulsivity is central to understanding the neurobiology of gambling and substance use disorders, in that they are characterized by maladaptive repetitive engagement in habitual behaviors that are reinforcing (implicating dysfunctional reward circuitry) (Grant & Chamberlain, 2016). Symptoms of dependence and withdrawal are extremely common in substance and gambling disorders, serving to perpetuate narrowing of the behavioral repertoire (Wareham & Potenza, 2010). While not yet regarded as a formal psychiatric disorder, problematic Internet use is relatively commonplace. Its working diagnostic criteria incorporate compulsive use (based on parallels with substance use disorders) (Young, 2009). Based on an extensive review of available literature, compulsive use has been highlighted as a core symptom of Internet addiction (Kuss *et al.* 2014). Compulsivity has emerged as a key construct in understanding eating disorders – pathological overeating (Degortes *et al.* 2014; Moore *et al.* 2017), as well as in anorexia nervosa (Tenconi *et al.* 2010; Degortes *et al.* 2014; Treasure *et al.* 2015). For compulsive personality, obsessive–compulsive symptom traits on the Padua Inventory revised (Burns *et al.* 1996; Sanavio, 1988), and OCPD traits (based on number of DSM criteria met), were quantified. We used Padua Inventory rather than the OCD Yale-Brown Obsessive Compulsive Disorder Scale (YBOCS) because the Padua Inventory is designed to explore obsessive–compulsive traits and symptoms at the population level; using the YBOCS in a normative population would likely have yielded very limited variation in scores, with most participants scoring zero. For compulsive cognition, we measured reversal learning and extra-dimensional set-shifting on the Intra-Dimensional/Extra-Dimensional Set-Shift Task (IED); along with risk adjustment on the Cambridge Gamble Task (CGT). Reversal learning and extra-dimensional set-shifting are two key, separable components of behavioral flexibility germane to understanding compulsivity (Clarke *et al.* 2005; Clarke *et al.* 2007; Chamberlain & Menzies, 2009; Dalley *et al.* 2011). It was hypothesized that difficulty adjusting behavior as a function of risk on the CGT would constitute a measure of decision-making sensitive to rigid response styles. See online Supplementary file for more detailed description of the cognitive tasks.

### Data analysis

All statistical analyses were undertaken using R software, MPlus, and IBM SPSS (v22.0) (Muthén & Muthén, 2016; R Core Team, 2016).

Impulsive and compulsive measures of interest were analyzed using confirmatory factor analysis (CFA) to compare three models: one in which impulsive and compulsive measures were underpinned by a single underlying latent factor; one in which covariances among impulsive and compulsive measures were explained by two underlying unrelated latent factors; and one in which covariances were explained by two underlying related latent factors. Our aim was to test the relationship between these latent factors, rather than to explore the possible existence of other additional latent factors. As such, and in view of the extensive literature supporting the existence of these two latent types of measurement (impulsivity and compulsivity) (Robbins *et al.* 2012; Guo *et al.* 2017), CFA rather than exploratory factor analysis was appropriate. We included behavioral, personality, and cognitive measures on the same conceptual plane to maintain simplicity of the examined models but also because the distinction between these categories of measure is far from clear. DSM-5 places personality disorders and disorders formally regarded as being on ‘axis-I’ on the same conceptual plane, in recognition of overlap between them. Many measures can be argued to be in one category or another depending on vantage point. To assure comparability, the model fit was evaluated using Akaike Information Criterion (AIC) and Bayesian Information Criterion (BIC) (Akaike, 1974; Schwarz, 1978).

## Results

**Fig. 1.**
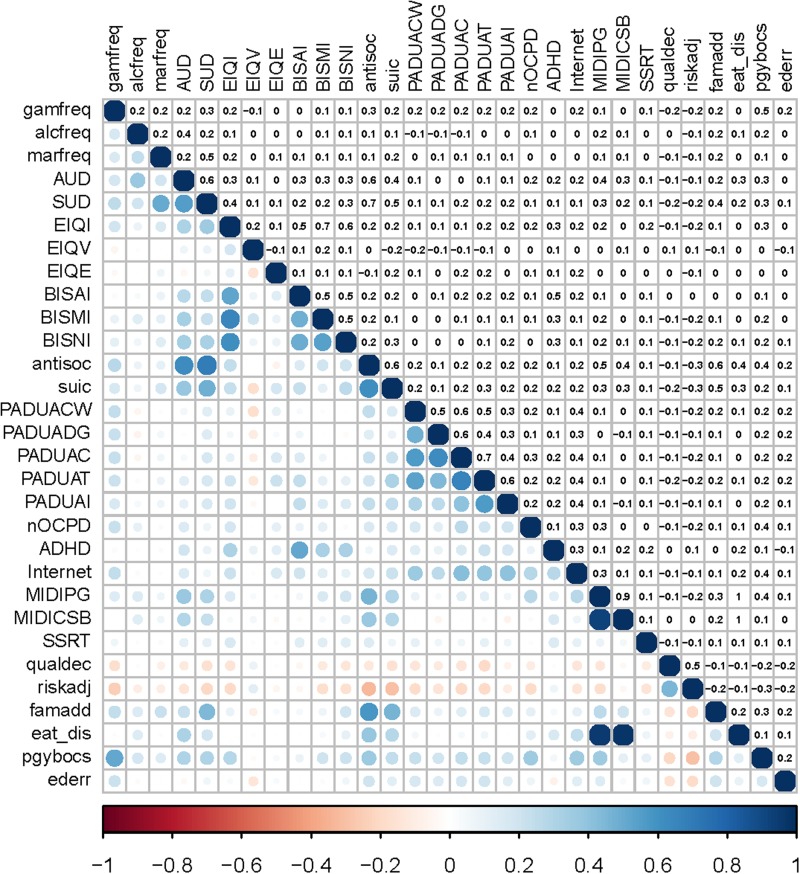
Heat map showing correlations between variables of interest. Left: positive correlations are shown in blue, and negative in red; larger dots are indicative of stronger correlations. Right: correlation coefficients (rounded to one decimal place in the interests of clarity). gamfreq, gambling frequency per week; alcfreq, alcohol frequency per week; marfreq, marihuana frequency per week; AUD, alcohol use disorder; SUD, substance use disorder; EIQI, Eysenck Inventory Impulsiveness; EIQV, Eysenck Inventory Venturesomeness; EIQE, Eysenck Inventory Extraversion; BISAI, Barratt Attentional Impulsiveness; BISMI, Barratt Motor Impulsiveness; BISNI, Barratt Non-Planning Impulsiveness; antisoc, antisocial personality disorder; suic, suicidality on the Mini International Neuropsychiatric Inventory; PADUACW, Padua Inventory Contamination and Washing subscale; PADUADG, Padua Inventory Dressing/Grooming subscale; PADUAC, Padua Inventory Checking subscale; PADUAT, Padua Inventory Thoughts of Harm to Self-others subscale; PADUAI, Padua Inventory Impulses to Harm Self/Others subscale; nOCPD, number of obsessive-compulsive personality disorder criteria met; ADHD, total score on attention-deficit hyperactivity disorder screen; Internet, total score on Young's Internet Addiction Test; MIDIPG, gambling disorder; SSRT, stop-signal reaction time on Stop-Signal Test; qualdec, quality of decision-making on the Cambridge Gamble Test; riskadj, risk adjustment on the Cambrige Gamble Test; eat_dis, eating disorder; pgybocs, Pathological Gambling Yale Brown Obsessive–Compulsive Disorder Scale; ederr, extra-dimensional errors on the set-shifting task.

The sample comprised 576 individuals [mean age (s.d.) = 22.3 (3.6) years; 65.5% male]. The average quality of life, Barratt impulsiveness scores, Padua obsessive–compulsive scores, and cognitive scores were similar to those reported in previous normative datasets (for further discussion and distribution of individual measures see online Supplementary file). Correlations between individual measures of interest are summarized in Fig. 1.

### Confirmatory factor analysis

Fit indices for the different confirmatory factor models are summarized in Table 2; it can be seen that the model with two correlated latent factors had the best fit (lowest information criteria scores). This model was re-estimated using weighted least squares to assess its absolute fit, which yielded parameters as follows: global fit index 0.977, root mean square error of approximation 0.064, and comparative fit index 0.86. These values are considered to indicate reasonably good fit, in view of conventional criteria, taking into consideration the sample size (Hu & Bentler, 2009). Comparing the hypothesized models using weight of evidence, the two-factor correlated model was the best fit (>99.9% probability).

**Table 2.** Maximum likelihood-based fit indices

Model	AIC	BIC	aBIC
One-factor	53 978	54 301	54 066
Two-factor uncorrelated	53 212	53 535	53 300
Two-factor correlated	53 186	53 513	53 275

Lower scores indicate better model fit, and a reduction of 10 or more units from one model to another is generally held to indicate a superior model (Raftery, 1995).

**Fig. 2.**
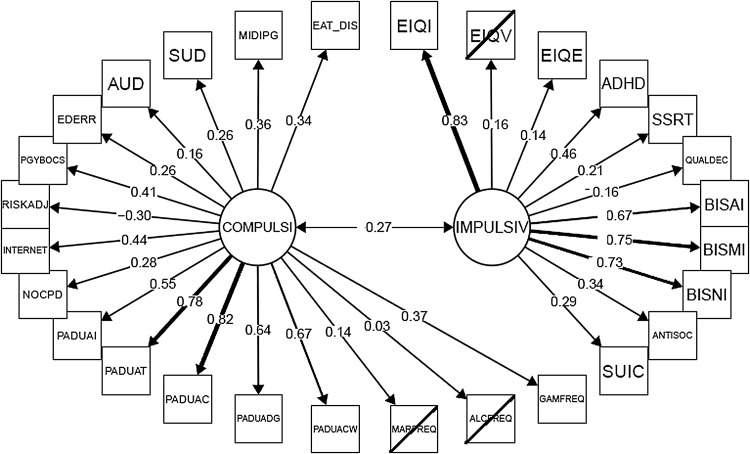
Two-factor correlated model, showing factor loadings. Variables with non-statistically significant loading (*p* > 0.05) are shown in strike-through. Abbreviations – see footer to Fig. 1.

The loadings of manifest measures onto the optimal two-factor model are shown in Fig. 2. Variable loadings were in the expected direction, such that higher impulsivity factor scores were associated with higher personality measures of impulsivity, higher impulsive symptomatology (ASPD, ADHD, suicidality), and worse quality of decision-making on the gambling task; and higher compulsivity factor scores were associated with higher personality measures of compulsivity (Padua Inventory), higher compulsive symptomatology (gambling, problematic Internet use, OCPD, substance use disorders), more extra-dimensional set-shifting errors on the set-shifting task, and less risk adjustment on the gambling task.

Higher scores on the impulsivity and compulsivity factors, respectively, were significantly correlated with worse quality of life on the Quality of Life Inventory (*r* = 0.27, *p* < 0.00001; and *r* = 0.30, *p* < 0.000001).

## Discussion

Mental disorders characterized by impulsive or compulsive symptoms constitute a huge burden for patients, family members, and society at large. Psychiatry is seeking methods of refining the definition of mental disorders, and of better understanding their etiology and neurobiological substrates. One way of tackling this challenge (Cuthbert & Insel, 2013), is to use a multi-tiered approach examining not just categorical mental disorders but also dimensional psychopathology, personality, and neurocognitive functioning (Chamberlain & Menzies, 2009). Here, we examined such multi-tiered measures in a sample of young adults who were not treatment seeking. We found that impulsive and compulsive manifest measures were underpinned by distinct latent traits of impulsivity and compulsivity, which were positively correlated with each other. Furthermore, higher scores on each of these latent traits were significantly correlated with worse quality of life, thereby confirming their clinical relevance. The two correlated factor model was a better fit to the data than alternative conceptual models in which impulsive and compulsive manifest measures were mediated by a single latent factor of ‘disinhibition’; and the alternative conceptualizations that impulsive and compulsive latent traits were in opposition to each other (negatively correlated) or independent of each other. We focused on impulsivity and compulsivity, which we view as being complementary to other suggested frameworks for understanding mental disorders, such as the idea of externalizing *v.* internalizing symptoms; or the existence of a bipolar mood spectrum. Impulsivity and compulsivity are related to these other models but are not the same thing (Eisenberg *et al.* 2009). Ultimately, psychiatry may benefit from a more cohesive model that integrates multiple of these ideas (Lara & Akiskal, 2006).

Manifest measures loading most strongly and significantly on the latent trait of impulsivity (see Fig. 2) were (in descending order of statistical significance): impulsiveness on the Eysenck Inventory, impulsiveness on the Barratt questionnaire, dimensional ADHD symptoms, presence of ASPD, suicide risk, longer stop-signal reaction times, and extraversion on the Eysenck Inventory. As a caveat, it should be noted that some of these loadings, while statistically significant, were relatively small in magnitude. These findings are consistent with studies in clinical populations, which have reported positive associations between such personality-based measures of impulsivity, and ADHD (Malloy-Diniz *et al.* 2007); and between such personality-based measures and ASPD (Swann *et al.* 2009). There is also evidence that Barratt impulsiveness was associated with suicidality in the context of depressive symptoms (Swann *et al.* 2008). Stop-signal reaction time impairment has been observed in meta-analysis of available cognitive studies comparing ADHD groups to healthy volunteers (Lijffijt *et al.* 2005; Chamberlain *et al.* 2011). In non-treatment-seeking adults with ASPD, stop-signal deficits were observed *v.* healthy controls (Heritage & Benning, 2013; Chamberlain *et al.* 2016).

In contrast to research regarding impulsivity, the exploration of compulsivity has received relatively little research attention (Robbins *et al.* 2012). Manifest measures of compulsivity that loaded significantly onto the latent factor of compulsivity were as follows (in descending order of statistical significance): obsessive–compulsive symptoms on the Padua Inventory, problematic Internet use, problem gambling symptoms, presence of an eating disorder, less risk adjustment on the CGT, OCPD traits, more extra-dimensional set-shift errors on the Set-Shift Task (IED), and presence of substance/alcohol use disorder. Again, some of these variables had relatively low loading onto the latent construct, albeit they were significant. Compulsivity has been defined, hypothetically, as an intermediate phenotype or trait characterized by persistence of habitual/repetitive actions despite untoward consequences (Robbins *et al.* 2012). Our data are supportive of the view that compulsivity can be conceptualized as such a trait, and demonstrate that it is largely separable from impulsivity. Relatively impaired set-shifting has been identified in patients with OCD (Veale *et al.* 1996; Watkins *et al.* 2005; Chamberlain *et al.* 2007), and in those with eating disorders (Kanakam & Treasure, 2013; Aloi *et al.* 2015; Perpina *et al.* 2016), compared with healthy controls. Set-shifting errors, and reduction of behavioral adjustment as a function of risk, are both indicative of inflexible or habitual response styles. One interpretative model is that these response patterns may be due to a tendency toward habitual behaviors at the expense of goal-directed behaviors (Gillan & Robbins, 2014). In a large-scale Internet-based study, deficits in goal-directed control were strongly associated with compulsive behaviors and intrusive thoughts, but were also associated to a lesser degree with impulsivity on the Barratt questionnaire (Gillan *et al.* 2017).

Problematic Internet use is not yet considered a mental disorder in DSM-5, but has been highlighted as a concept in need of further study (in this and other narrower guises, such as ‘Internet Gaming Disorder’) (Grant & Chamberlain, 2016). Studies report high rates of mental disorders including OCD in people with pathological Internet use (Carli *et al.* 2013; Durkee *et al.* 2016). In an Internet-based survey, some types of obsessive–compulsive symptoms (impulses to harm self/others and checking compulsions) were the most important variables across impulsive–compulsive measures in terms of classifying participants as having moderate–severe Internet addiction (Ioannidis *et al.* 2016). Research into OCPD is scant, but data indicate disproportionately worse set-shifting impairment in OCD patients with this comorbidity (Fineberg *et al.* 2007; Fineberg *et al.* 2015). Our finding that substance use disorders loaded significantly and positively onto the latent compulsivity factor is unsurprising. Criteria for substance use disorders includes features such as narrowing of the behavioral repertoire, persistently engaging in use despite negative consequences, and unsuccessful attempts to cut back, which fit the concept of compulsivity closely.

To our knowledge, no previous studies have explored the latent structure of impulsivity and compulsivity measures within one setting, using such a broad range of measures. However, there is an extensive body of literature, mostly focusing on psychopathology, suggesting the existence of an underlying ‘internalizing’ dimension (predisposition to anxiety/depressive disorders) and an ‘externalizing’ dimension (predisposition to, e.g. substance use, antisocial personality, ADHD) (Caspi *et al.* 2014). OCD symptoms have not been consistently included in studies exploring the structure of psychopathology (Caspi *et al.* 2014). In a large sample of adolescents (IMAGEN consortium), the best-fit model for selected psychopathological measures comprised two factors (compulsivity and externalizing behaviors) (Montigny *et al.* 2013). The compulsivity factor showed strong associations with OCD and eating disorders, whereas the externalizing behaviors factor showed strong associations with substance misuse, conduct disorder, and (to a lesser degree) ADHD symptoms. In another paper by the same research consortium (IMAGEN), psychopathological measures were first entered into initial CFA (Castellanos-Ryan *et al.* 2016). The relationships between resulting factors and other measures, such as cognitive functioning were then explored. The authors opted for a bi-factor model incorporating three factors. It was found that a general psychopathology factor correlated with worse response inhibition and greater temporal discounting; an externalizing factor correlated with high risk taking on a gambling task; and that an internalizing factor correlated with attentional bias toward negatively valenced verbal stimuli. This study measured impulsivity with a go/no-go task (rather than a stop-signal task), and did not quantify attentional set-shifting.

The current study has several limitations, which merit consideration. Our sample did not exclude people with mental disorders. This can be viewed as being beneficial to exploring latent traits of impulsivity and compulsivity since it would have yielded a broader set of data for the purposes of measuring covariance, and is also parsimonious with the RDoc concept. Our sample showed average impulsive and compulsive personality questionnaire scores, and cognitive performance, akin to that reported in previous normative populations. However, the findings may not generalize to other populations, such as patients in clinical settings. We treated personality, symptoms, and cognition as being on the same conceptual plane, with a view to maintaining model simplicity and avoiding unnecessary assumptions. We used confirmatory rather than exploratory factor analysis because the concepts of impulsivity and compulsivity are well established in the literature (Hollander & Cohen, 1996; Robbins *et al.* 2012) and our research question pertained to the relationship between them, rather than the separate issue of whether more latent factors exist. For pragmatic reasons, we did not include all possible measures of relevance to impulsivity and compulsivity. For example, we did not assess reflection–impulsivity, or tasks of ‘incremental’ habit learning (Gillan & Robbins, 2014; Hauser *et al.* 2016). It would be valuable to include such parameters in future work. Lastly, some recent studies of personality and psychopathology have explored bi-factor models. In bi-factor models, a general factor or ‘*p*’ (analogous to the historical ‘*g*’ factor in intelligence quotient research) is included, on the assumption that a proportion of covariance across all measures may measure a common trait (Caspi *et al.* 2014). The findings from the two correlated factor model suggest against using a bi-factor structure for the current data, because the proportion of shared variance between the impulsivity and compulsivity factors was only ~7%. Furthermore, bi-factor models may be intrinsically biased toward yielding superior model fit parameters over non bi-factor models, as a consequence of unmodeled complexity (Murray & Johnson, 2013). In *post hoc* analysis, furthermore, we found that the *ω* coefficient was inferior when a bi-factor model fit was applied to the data (*ω* < 0.7 for the general factor), *v.* the two correlated factor model (*ω*_impulsivity_ = 0.74; *ω*_compulsivity_ = 0.79) (Reise, 2012). The biological and clinical plausibility and interpretation of ‘*g*’ or ‘*p*’ factor may be problematic (Vanheule *et al.* 2008) – for example, ‘*p*’ could represent response style rather than containing substantial meaning.

In summary, this study used a range of psychopathological, personality, and cognitive measures to demonstrate the existence of two separable but positively correlated latent traits of impulsivity and compulsivity, in a sample of young adults recruited from the background population. Higher scores on each latent trait was significantly correlated with worse quality of life, and was associated with greater risk of having one or more relatives with a behavioral or substance addiction, supporting the clinical relevance of these traits.

We do not suggest that all manifest measures of impulsivity and compulsivity are ‘two things’; there is evidence, for example, that different measures of impulsivity can be fractionated (Meda *et al.* 2009; MacKillop *et al.* 2016). However, the current findings do support the notion that latent forms of impulsivity and compulsivity exist in a dimensional or continuous sense that they are largely separable from each other, and that higher impulsivity tends to associate with higher compulsivity (rather than the opposite). Treatments targeting impulsivity and compulsivity at the conceptual level, or in terms of specific manifest forms (such as neurocognitive impairment), would be potentially valuable. Future work should identify neural and neurochemical underpinnings of these latent dimensions with a view to informing nosological and neurobiological models; and should also attempt to integrate multiple candidate dimensions besides impulsivity and compulsivity.

## Supplementary material

For supplementary material accompanying this paper visit http://dx.doi.org/10.1017/S0033291717002185. click here to view supplementary material
