# Supplementary material for: Latent traits of impulsivity and compulsivity: toward dimensional psychiatry
Source: Psychol Med. 2017 Aug 14;48(5):810–21. doi: 10.1017/S0033291717002185 (PMC5699644; doi:10.1017/S0033291717002185)
Supplement: Supplementary file 1 [file S0033291717002185sup001.docx]

Online Supplementary File

Chamberlain et al.

**Comparison of key sample features to normative data reported previously**

The mean quality of life t-score was 45.9 (12.1), which is in the normal range for adults (cut-off for low quality of life is ~37 or below) {Frisch, 2005 #4799;Frisch, 1998 #4804}. Mean Stop-Signal, set-shifting, and decision-making performances were similar to normative data reported in previous studies using healthy controls free from mental disorders e.g. {Chamberlain, 2007 #4460}. Mean Barratt Impulsiveness scores very alike normative data reported elsewhere: attention mean 16.9 [4.1] versus 17.2 [3.9] in {Spinella, 2007 #7736}; motor 23.8 (4.7) versus 22.1 [4.4]; non-planning 24.2 [5.3] versus 24.9 [5.1]. Mean Padua scores were also comparable to previously reported normative data: contamination/washing score mean 6.2 [7.0] versus 6.5 [5.5] in {Burns, 1996 #2254}; dressing/grooming score 1.4 [2.4] versus 2.0 [2.5]; checking 6.7 [6.5] versus 7.5 [6.4]; thoughts about harm to self/others 2.2 [3.3] versus 2.9 [3.5]; impulses to harm self/others 1.7 [3.4] versus 2.8 [4.1].

**Description of cognitive tasks**

Impulsive cognition was measured using the Stop-Signal Task (SST) and measures from the Gambling Gamble Task (CGT). On the SST {Aron, 2007 #2187;Logan, 1984 #315}, participants responded to directional arrows appearing on the computer screen, and had to attempt to suppress their responses whenever a stop-signal (auditory ‘beep’) occurred. The outcome measure of interest was the stop-signal reaction time, which was an estimate of the time taken by the given volunteer’s brain to suppress a response that would normally be undertaken. On the CGT {Rogers, 1999 #1301}, for each trial, participants gambled a proportion of their cumulative points on whether the computer had hidden a token behind a red or blue box. By varying the proportions of red and blue boxes, and the method by which the gambling amount was selected, the task enabled different aspects of decision-making to be captured. The key impulsive outcome measures for the CGT was quality of decision-making (the proportion of trials where the volunteer chose red when red boxes were in the majority and vice versa – i.e. made the logical color choice).

Cognitive compulsivity was measured using the risk adjustment measure from the previously described CGT task (tendency to adjust how many points are gambled depending on the degree of risk) and by the number of extra-dimensional shift errors on the Intra-Dimensional/Extra-Dimensional Set-shift task (IED) {Owen, 1991 #1299}. For the IED task, participants attempted, through trial and error, to work out an underlying ‘rule’ about which of two stimuli presented on the computer screen was correct. The computer had chosen this rule in advance. Once the individual had learnt the current rule, the computer changed it, enabling different aspects of learning and flexible responding to be measured. On the crucial extra-dimensional shift stage, the volunteer must ‘unlearn’ the previously relevant stimulus dimension and refocus their attention to a previously irrelevant stimulus dimension.

**Table 1. Data distributions for individual measures.**

| Variable | | Mean | | SD | | SE | |  |  |  |  |  |
| --- | --- | --- | --- | --- | --- | --- | --- | --- | --- | --- | --- | --- |
| gamfreq | | 1.76 | | 2.09 | | 0.09 | |  |  |  |  |  |
| alcfreq | | 1.43 | | 1.47 | | 0.06 | |  |  |  |  |  |
| marfreq | | 0.72 | | 1.76 | | 0.07 | |  |  |  |  |  |
| nicquan | | 0.13 | | 0.3 | | 0.01 | |  |  |  |  |  |
| Bmi | | 24.64 | | 7.42 | | 0.31 | |  |  |  |  |  |
| EIQI | | 7.95 | | 4.29 | | 0.18 | |  |  |  |  |  |
| EIQV | | 10.5 | | 3.42 | | 0.14 | |  |  |  |  |  |
| EIQE | | 12.18 | | 3.55 | | 0.15 | |  |  |  |  |  |
| BISAI | | 16.86 | | 4.07 | | 0.17 | |  |  |  |  |  |
| BISMI | | 23.81 | | 4.66 | | 0.19 | |  |  |  |  |  |
| BISNI | | 24.21 | | 5.33 | | 0.22 | |  |  |  |  |  |
| PADUACW | | 6.18 | | 7 | | 0.29 | |  |  |  |  |  |
| PADUADG | | 1.43 | | 2.41 | | 0.1 | |  |  |  |  |  |
| PADUAC | | 6.71 | | 6.52 | | 0.27 | |  |  |  |  |  |
| PADUAT | | 2.21 | | 3.25 | | 0.14 | |  |  |  |  |  |
| PADUAI | | 1.74 | | 3.37 | | 0.14 | |  |  |  |  |  |
| nOCPD | | 1.17 | | 1.53 | | 0.09 | |  |  |  |  |  |
| ADHD | | 9.17 | | 4.58 | | 0.22 | |  |  |  |  |  |
| Internet | | 2.43 | | 2.17 | | 0.15 | |  |  |  |  |  |
| SSRT | | 181.9 | | 64.24 | | 2.68 | |  |  |  |  |  |
| delav | | 0.3 | | 0.25 | | 0.01 | |  |  |  |  |  |
| propbet | | 0.54 | | 0.14 | | 0.01 | |  |  |  |  |  |
| qualdec | | 0.95 | | 0.09 | | 0 | |  |  |  |  |  |
| riskadj | | 1.55 | | 1.21 | | 0.05 | |  |  |  |  |  |
| age | | 22.27 | | 3.57 | | 0.15 | |  |  |  |  |  |
| qolit | | 45.85 | | 12.11 | | 0.51 | |  |  |  |  |  |
| pgybocs | | 5.14 | | 6.26 | | 0.26 | |  |  |  |  |  |
| reverr | | 6.18 | | 5.76 | | 0.24 | |  |  |  |  |  |
| ederr | | 10.28 | | 10 | | 0.42 | |  |  |  |  |  |
| Frequencies (for categorical type of variables) | | | | | | | | | |  |  |  |
|  | | 0 | | 1 | | 2 | | 3 | 4 | 5 | 6 | 7 |
| AUD | | 443 | | 130 | | - | | - | - | - | - | - |
| SUD | | 495 | | 81 | | - | | - | - | - | - | - |
| antisoc | | 544 | | 32 | | - | | - | - | - | - | - |
| suic | | 474 | | 85 | | 8 | | 9 | - | - | - | - |
| MIDICB | | 471 | | 23 | | - | | - | - | - | - | - |
| MIDIPG | | 401 | | 94 | | - | | - | - | - | - | - |
| MIDICSB | | 481 | | 14 | | - | | - | - | - | - | - |
| gender | | 199 | | 377 | | - | | - | - | - | - | - |
| marital | | - | | 511 | | 22 | | 7 | 35 | - | - | - |
| edu | | - | | 22 | | 43 | | 359 | 102 | 50 | - | - |
| emp | | - | | 115 | | 63 | | 198 | 69 | 1 | 128 | 1 |
| race | | - | | 422 | | 85 | | 19 | 36 | 6 | 1 | - |
| dep | | 560 | | 13 | | - | | - | - | - | - | - |
| anymini | | 365 | | 211 | | - | | - | - | - | - | - |
| famadd | | 396 | | 180 | | - | | - | - | - | - | - |
| eat_dis | | 475 | | 19 | | - | | - | - | - | - | - |

**References**

**Aron AR, Durston S, Eagle DM, Logan GD, Stinear CM , Stuphorn V** (2007). Converging evidence for a fronto-basal-ganglia network for inhibitory control of action and cognition. *Journal of Neuroscience* **27**, 11860-4.

**Burns GL, Keortge SG, Formea GM , Sternberger LG** (1996). Revision of the Padua Inventory of obsessive compulsive disorder symptoms: distinctions between worry, obsessions, and compulsions. *Behaviour Research and Therapy* **34**, 163-73.

**Chamberlain SR, Fineberg NA, Menzies LA, Blackwell AD, Bullmore ET, Robbins TW , Sahakian BJ** (2007). Impaired cognitive flexibility and motor inhibition in unaffected first-degree relatives of patients with obsessive-compulsive disorder. *Am J Psychiatry* **164**, 335-8.

**Frisch MB** (1998). Quality of life therapy and assessment in health care. *Clinical Psychology: Science and Practice* **5**, 19-40.

**Frisch MB, Clark MP, Rouse SV, Rudd MD, Paweleck JK, Greenstone A , Kopplin DA** (2005). Predictive and treatment validity of life satisfaction and the quality of life inventory. *Assessment* **12**, 66-78.

**Logan GD, Cowan WB , Davis KA** (1984). On the ability to inhibit simple and choice reaction time responses: a model and a method. *Journal of Experimental Psychology: Human Perception and Perforamance* **10**, 276-91.

**Owen AM, Roberts AC, Polkey CE, Sahakian BJ , Robbins TW** (1991). Extra-dimensional versus intra-dimensional set shifting performance following frontal lobe excisions, temporal lobe excisions or amygdalo-hippocampectomy in man. *Neuropsychologia* **29**, 993-1006.

**Rogers RD, Everitt BJ, Baldacchino A, Blackshaw AJ, Swainson R, Wynne K, Baker NB, Hunter J, Carthy T, Booker E, London M, Deakin JF, Sahakian BJ , Robbins TW** (1999). Dissociable deficits in the decision-making cognition of chronic amphetamine abusers, opiate abusers, patients with focal damage to prefrontal cortex, and tryptophan-depleted normal volunteers: evidence for monoaminergic mechanisms. *Neuropsychopharmacology* **20**, 322-39.

**Spinella M** (2007). Normative data and a short form of the Barratt Impulsiveness Scale. *International Journal of Neuroscience* **117**, 359-68.
